# Innovative Therapy with Stem Cell-Derived Extracellular Vesicles on Cardiac Hypertrophy in an Animal Model of Atherosclerosis; Elucidation of the Molecular Mechanisms Involved in the Repair Process

**DOI:** 10.3390/biom15101424

**Published:** 2025-10-07

**Authors:** Alexandra Vîlcu, Ioana Karla Comarița, Alina Constantin, Nicoleta Alexandru, Miruna Nemecz, Florentina Safciuc, Florina Bojin, Virgil Păunescu, Adriana Georgescu

**Affiliations:** 1 Department of Pathophysiology and Cellular Pharmacology, Institute of Cellular Biology and Pathology ‘Nicolae Simionescu’ of Romanian Academy, 050568 Bucharest, Romania; vilcu.alexandra18@gmail.com (A.V.); karla.comarita@icbp.ro (I.K.C.); alina.constantin@icbp.ro (A.C.); nicoleta.alexandru@icbp.ro (N.A.); miruna.nemecz@icbp.ro (M.N.); florentina.safciuc@icbp.ro (F.S.); 2 Immuno-Physiology and Biotechnologies Center (CIFBIOTECH), Department of Functional Sciences, “Victor Babes” University of Medicine and Pharmacy, 300041 Timisoara, Romania; florinabojin@umft.ro (F.B.); vpaunescu@umft.ro (V.P.); 3 Center for Gene and Cellular Therapies in the Treatment of Cancer Timisoara-OncoGen, Clinical Emergency County Hospital “Pius Brinzeu” Timisoara, 300723 Timisoara, Romania

**Keywords:** atherosclerosis, cardiac hypertrophy, inflammation, extracellular vesicles, mesenchymal stem cells, Smad2/3 siRNA

## Abstract

**(1)** Background: The present study investigated the effects of extracellular vesicles (EVs), derived from adipose tissue stem cells (ADSCs) and bone marrow mesenchymal stem cells (BMMSCs), on atherosclerosis-associated cardiac hypertrophy. **(2)** Methodology: The experiments were performed on hamsters divided into the following groups: control (C) fed with a standard diet; hypertensive–hyperlipidemic (HH) generated by combining a diet enriched with 3% cholesterol, 15% butter, and by gavage with 8% NaCl on a daily basis; HH groups injected with EVs (ADSCs) or EVs (BMMSCs), either transfected with Smad2/3 siRNAs or not (HH-EVs (ADSCs), HH-EVs (BMMSCs), HH-EVs (ADSCs) + Smad2/3siRNA, HH-EVs (BMMSCs) + Smad2/3siRNA); and HH group injected with Smad2/3 siRNAs (HH-Smad2/3siRNA). **(3)** Results: In comparison with the HH group, the findings demonstrated that treatment using EVs (ADSCs or BMMSCs), either with or without Smad2/3 siRNAs, resulted in several significant improvements in the following aspects: the plasma levels of cholesterol, LDL, triglycerides, TGF-β1, and Ang II were decreased; the left ventricular structure and function were recovered; inflammatory markers, ROS, COL1A, α-SMA, Cx43, MIF, ANF, and M1/M2 macrophages, were reduced; the level of key protein NF-κB p50 was diminished. **(4)** Conclusions: These findings underscore the therapeutic potential of mesenchymal stem cell-derived EVs in atherosclerosis-associated cardiac hypertrophy.

## 1. Introduction

Atherosclerosis is the leading cause of cardiovascular disease, with the World Health Organization (WHO) estimating that about 31% of deaths worldwide are caused by it. Atherosclerosis is a progressive disease that is characterized by the narrowing of the lumen of blood vessels due to the deposition of cholesterol plaques [1].

Studies carried out over time on experimental animals have aimed to elucidate the stages of this pathology but also the molecular mechanisms that trigger it. These studies have shown that vascular endothelium plays a key role in regulating the permeability of blood vessels, being the one that makes the connection between the risk factors and the initiation of the atherosclerotic process [2]. The crucial event in triggering the atherogenic process is the oxidation of low-density lipoprotein (LDL) cholesterol molecules. Thus, under the influence of risk factors such as excess LDL in the blood, hypertension, diabetes mellitus etc., the vascular endothelium becomes more permeable to LDL that will enter the intima [3]. At this point, the endothelial cells (ECs) from the intima respond to the presence of the lesion by secreting adhesion molecules including ICAM-1 (intercellular adhesion molecule-1) and VCAM-1 (vascular-cell adhesion molecule-1), which will increase the adhesion properties of the vascular endothelium. Moreover, both smooth-muscle cells (SMCs) and ECs will secrete MCP-1 (monocyte chemotactic protein-1) and M-CSF (monocyte-colony stimulating factor), compounds that cause the recruitment of monocytes in the vascular endothelium. At this level, the monocytes turn into macrophages that generate reactive oxygen species that will oxidize LDL, resulting in oxidized LDL (ox-LDL) [4]. Afterward, the macrophages that have taken over ox-LDL become overloaded with lipids and turn into foam cells. These, in turn, will release lipids into the intima and migrate to the lumen of the arterial wall. Ultimately, these lipids gradually accumulate, forming the center of an atherosclerotic plaque, the so-called lipid pool. In addition, the foam cells secrete cytokines such as PDGF (platelet-derived growth factor), IL-1 (interleukin-1), IFN-γ (interferon-gamma), TNF-α (tumor necrosis factor-alpha), and TGF-β (transforming growth factor-beta) that stimulate SMC proliferation and migration to the lumen of the arterial wall [5]. At this level, SMCs will synthesize extracellular matrix proteins, in particular collagen and fibronectin, which will be stored in the forming atherosclerotic plaque, resulting in a fibrous cap over the lipid core. At this point, the structure of the arterial wall is compromised, and the fibro-atheromatous plaque enters the lumen of the artery, obstructing the blood flow [6]. Over time, atherosclerotic plaque becomes vulnerable to rupture because macrophages and T lymphocytes that are localized preferentially at the edges of the plaque secrete matrix metalloproteinases (MMP) that degrade extracellular matrix proteins, and inhibit SMC proliferation and collagen synthesis which weakens the fibrous cap [7]. Moreover, the SMCs present in the most vulnerable regions of the plaque undergo apoptosis, the event that favors the rupture of the atherosclerotic plaque. The rupture of the atherosclerotic plaque causes bleeding and draws platelets to this level, initiating a coagulation process with the formation of an obstructive thrombus at the rupture site. Under the influence of blood flow, the thrombus may detach, leading to thromboembolic processes such as myocardial infarction or stroke [8].

We can thus unequivocally state that atherosclerosis is an extremely complex disease, its complexity being doubled by the fact that it is often associated with other pathologies. One of these associated pathologies is cardiac hypertrophy. Without a clear connection between these two established yet, more common points between these pathologies were identified. Firstly, atherosclerosis and cardiac hypertrophy have a number of shared risk factors. These include smoking, obesity, ageing, and, most significantly, hypertension. Specifically, in hypertensive conditions, the myocardium must respond to hemodynamic overload, under constant mechanical stress. Therefore, the left ventricle must generate higher wall tension, triggering concentric hypertrophy and chamber dilatation [9]. In terms of the connection with atherosclerosis from this perspective, one of the observations refers to the stiffening of the arteries in conditions of hypertension and ageing [10]. These represent another cause for the development of cardiac hypertrophy, especially of the left ventricle due to pulsatile hemodynamic alterations. In relation to the molecular mechanisms and the parallels that exist between the etiology of the two pathologies, a number of markers, cell types, and signaling pathways have been identified. One of the mechanisms involved is the renin–angiotensin system. Ang II (Angiotensin II) has been demonstrated to induce a pro-inflammatory response in the arteries, regulating the expression of cytokines and chemokines. In SMCs, Ang II has been demonstrated to induce the transcriptional activation of NF-κB (Nuclear Factor Kappa B) and the expression of pro-inflammatory molecules, including IL-6 and MCP-1 [11]. Ang II has been demonstrated to activate the intracellular signaling pathway known as MAPK (Mitogen-Activated Protein Kinase), which has been shown to stimulate the growth of cardiomyocytes [12]. Another mechanism involves TGF-β, which is secreted by macrophages and vascular smooth-muscle cells in the atheromatous plaque. The process of binding of TGF-β1 to its receptors on cardiomyocytes and fibroblasts occurs concomitantly with its own autophosphorylation. Phosphorylated TGF-β1 activates SMAD proteins, which in turn overexpresses genes involved in extracellular matrix production, thus promoting cardiac remodeling [13].

Cardiac hypertrophy is an adaptation of the heart to increase because of hemodynamic stress, an adaptation based on the capacity of cardiomyocytes to increase in size [14]. Cardiac hypertrophy is considered pathological when the following processes are also highlighted in addition to an increase in the size of cardiomyocytes and protein synthesis: cellular fibrosis, cardiomyocyte apoptosis, calcium homeostasis disorder, mitochondrial dysfunction, reactivation of fetal genes, impaired sarcomere structure, and reduced angiogenesis. [15]. Specifically, the increase in blood flow pressure causes the secretion of cytokines and growth factors that promote the increase in the size of cardiomyocytes, the left ventricle being the most affected [16]. One of the key molecules that can tip the scales toward triggering hypertrophy is TGF-β. Specifically, TGF-β binds to its receptors that will activate SMAD (suppressor of mothers against decapentaplegic) proteins. The TGF-β-SMAD complex is translocated into the nucleus where it binds to the promoter regions of specific genes, thus expressing its effects. Under conditions of hypertension, these involve the induction of cardiomyocyte apoptosis and cardiac fibrosis, thus promoting the onset of cardiac hypertrophy [17]. Another significant molecule involved in cardiac hypertrophy is Nf-kb. NF-κB represents a family of transcription factors involved in a series of processes such as inflammation, immunity, proliferation, differentiation, and cell survival. The mammalian NF-κB family consists of the following proteins: RelA (p65), RelB, c-Rel, p105/p50 (NFKB1), and p100/p52 (NFKB2), that assemble into various transcriptionally homo- and heterodimeric complexes [18]. From these, the p50 and p52 derive from processing the precursor molecules p105 and p100 [19]. Usually, NF-κB dimers are retained in the cytoplasm by inhibitory IκB proteins. Upon stimulation, the IKK complex phosphorylates IκB, triggering its ubiquitination and proteasomal degradation; this frees NF-κB to translocate into the nucleus and activate target genes. NF-κB signaling proceeds through two principal routes: the canonical pathway, defined by IKK-dependent IκBα degradation and typically involving RelA (p65):p50 dimers, and the non-canonical pathway, driven by NIK–IKKα-mediated processing of p100 to p52, which activates p52:RelB complexes. Once in the nucleus, NF-κB activity is regulated by extensive post-translational modifications—phosphorylation, ubiquitination, and acetylation—that shape DNA binding, cofactor recruitment, and crosstalk with other signaling cascades [20]. Another aspect that has gained interest in recent years is that of extracellular vesicles (EVs). Given their involvement in intercellular communication, they are involved in a pleiade of physiological processes, whether normal or pathological. Moreover, it seems that under pathological conditions, their levels in the circulation and in organs/tissues rise, and they can be released from cardiomyocytes and other heart cell types like ECs or SMCs [21,22]. Therefore, these have been studied in the context of various diseases, such as diabetes, cancer, and cardiovascular disease as biomarkers or, on the contrary, as potential therapeutic strategies, most experiments targeting their content in microRNAs (miRNAs) [23,24,25].

According to their origin and typical dimensions, EVs can be classified into three distinct types: exosomes, microvesicles, and apoptotic bodies [26]. Of these three, the study of exosome and microvesicles is particularly extensive, with numerous studies providing evidence of their involvement in various pathological processes, including atherosclerosis [27,28]. However, the involvement of apoptotic bodies, in cardiovascular diseases, has not been highlighted [29]. EVs are involved in many cellular processes such as inflammation, signal transduction, thrombosis, immune response, cell proliferation, migration, and apoptosis due to their capacity to transport different molecules like enzymes, proteins, lipids, DNA, and various types of RNA. Therefore, it may be concluded that EVs play a pivotal role in facilitating cell-to-cell communication [30,31,32].

Regarding the involvement of the EVs in the development of the atherosclerotic process, they have been reported to be present in both intimal lesions in developing plaques and in advanced plaques, suggesting that they participate in the initial and final stages of plaque formation in humans. As demonstrated in the experiments conducted by Leroyer et al., 2007 [33], within fibro-atheromatous plaques, the prevalence of EVs derived from leukocytes is approximately 52%, with macrophage-derived EVs accounting for around 29%, lymphocyte-derived for 15%, and granulocyte-derived for approximately 8%. In addition, 13% of the EVs originate from SMCs and 8% from ECs [33].

The subsequent inquiry pertains to the function of these vesicles in the progression from endothelial dysfunction to the development of atheroma plaques. Recent studies have demonstrated that these vesicles are implicated in multiple processes intrinsic to atherosclerosis, encompassing lipid metabolism, endothelial dysfunction, inflammation, angiogenesis, vascular calcification, and thrombosis [34].

In view of the findings outlined above, the present study investigated the therapeutic potential of EVs derived from subcutaneous adipose tissue stem cells (ADSCs) or bone marrow mesenchymal stem cells (BMMSCs), either transfected or untransfected with a specific small interfering RNA (siRNA) designed to target Smad2/3 (Smad2/3 siRNAs), on cardiac hypertrophy in an animal model of human atherosclerosis. Specifically, by recreating the risk conditions for the development of cardiac hypertrophy associated with atherosclerosis, we first followed the changes that occur at the functional, structural, and molecular level in the left ventricle, mediated by Ang II and TGF-β1 via SMAD2/3, and NF-kB signaling molecules. In the subsequent stage of the study, a comparative analysis was conducted to investigate the therapeutic potential of Smad2/3 siRNA alone. This analysis involved re-examining the same changes and molecular mechanisms associated with cardiac hypertrophy.

## 2. Materials and Methods

### 2.1. Obtaining the Experimental Model of Cardiac Hypertrophy, Including the One Treated with EVs (ADSCs or BMMSCs), Transfected or Not with Smad2/3 siRNA

A group of 3-month-old *Golden Syrian* male hamsters (n = 67) was divided into seven experimental groups: (1) control group (C): healthy hamsters fed with a standard diet containing 1% NaCl (n = 11), (2) hypertensive–hyperlipidemic (HH) group fed with atherogenic diet consisting of standard food enriched with 3% cholesterol and 15% butter to induce hyperlipemia and daily gavage with 8% NaCl to induce hypertension (n = 18), (3) HH group treated with EVs produced by adipose tissue stem cells (HH-EVs (ADSCs)) (n = 10), (4) HH group treated with EVs produced by bone marrow mesenchymal stem cells (HH-EVs (BMMSCs)) (n = 9), (5) HH group treated with EVs from ADSCs transfected with Smad2/3 siRNA (HH-EVs (ADSCs) + Smad2/3siRNA) (n = 5), (6) HH group treated with EVs from BMMSCs transfected with Smad2/3siRNA (HH-EVs (BMMSCs) + Smad2/3siRNA) (n = 6), and (7) HH group treated with Smad2/3siRNA (HH-Smad2/3siRNA) (n = 8).

The seven groups were monitored over a period of four months. In the case of the treated groups (HH-EVs (ADSCs), HH-EVs (BMMSCs), HH-EVs (ADSCs) + Smad2/3siRNA, and HH-EVs (BMMSCs) + Smad2/3siRNA), the atherogenic diet (3% cholesterol and 15% butter) was administered and gavage with 8% NaCl was conducted over a period of two weeks. In the subsequent phase, the hamsters were injected with 100 µg/mL EVs (ADSCs) or 100 µg/mL EVs (BMMSCs) into the retro-orbital sinus once per month, in a volume of 300 µL phosphate-buffered saline (PBS), in a group-specific manner. The EVs (either ADSCs or BMMSCs) were either subjected to transfection with 100 nM Smad2/3 siRNAs or left untransfected. For the HH-Smad2/3 siRNA group, the injection was administered subcutaneously with 100 nM Smad2/3 siRNA in a volume of 300 µL PBS.

At the end of the four months of diet and treatment, the animals were sacrificed under anesthesia (a mixture of 80 mg ketamine, 10 mg xylazine, and 2 mg acepromazine/kg body weight in a sterile isotonic saline administered subcutaneously), the hearts were harvested and weighed. The left ventricles were taken to investigate the molecular mechanisms of interest in cardiac hypertrophy in atherosclerosis.

*Note*: All the animal use protocols were approved by the Ethics Committee from the Institute of Cellular Biology and Pathology “Nicolae Simionescu” according to Decision no.11/08.08.2017 and National Sanitary Veterinary and Food Safety Authority (Bucharest, Romania) in compliance with Project Authorization no. 575/13.11.2020. Also, all experiments on animals were conducted following the Guide of the Care and Use of Laboratory Animals published by the US National Institutes of Health (NIH publication no. 85-23, revised 1996) and were conducted following National, European, and International legislation on the use of experimental animals in biomedical research.

### 2.2. Obtaining and Characterizing the MSCs

EVs were isolated from stem cells produced by the adipose tissue and bone marrow, respectively. Therefore, in the initial phase of the study, the isolation of stem cells from these two distinct types of tissues was the primary focus.

For this, healthy male hamsters were sacrificed and the subcutaneous adipose tissue and bone marrow from the lower limb area were harvested. The adipose tissue was then trimmed and washed in ice-cold PBS to remove blood and impurities. Following this, collagenase was added, and the mixture was incubated for one hour at 37 °C. Following this step, 10% FBS (Fetal Bovine Serum) was added to neutralize collagenase activity. The samples were then spun at 400× *g* for 10 min, after which the upper layer was removed and the cell pellet was resuspended in erythrocyte lysis buffer for a further 10 min. The centrifugation process was then repeated at 400× *g* for 10 min at 4 °C, after which the cell pellet obtained was resuspended in DMEM/F-12 culture medium (Dulbecco’s Modified Eagle Medium/Nutrient Mixture F-12, Gibco, Waltham, MA, USA) with 10% FBS, supplemented with 1% antibiotic mix (a mixture of penicillin, neomycin, and streptomycin). A new round of centrifugation at 400× *g* for 10 min was applied, and then the pellet was passed through successive 100 µM and 70 µM filters and finally placed in the culture plate.

For BMMSCs, the femur and tibia were extracted, ensuring the epiphyses remained intact, and then placed in a Petri dish with cold PBS, along with a mixture of antibiotics and antifungals, which were kept on ice. The muscles were then removed from the bones, which had been thoroughly cleaned, using sterile compresses. The epiphyses were then cut with scissors. The procedure involved the use of a 26G syringe and a specialized cold BMMSC medium (containing DMEM medium with a high glucose concentration (1 g/L D-glucose), GlutaMAX™ Supplement, pyruvate (Gibco, Waltham, MA, USA), with 10% FBS for BMMSCs, supplemented with 1% antibiotic mix—a mixture of penicillin, neomycin, and streptomycin). The marrow was then removed from the medullary canal, and an 18G syringe was used to obtain a cell suspension. This was then subjected to a centrifugation process at 400× *g* for 5 min at 4 °C. The cell pellet was then subjected to erythrocyte lysis. The cells were counted and seeded at a density of 2 × 10^6^/cm^2^ on flasks that had been treated with 0.1% gelatin.

Both ADSCs and BMMSCs were cultured at 37 °C in 5% CO_2_ until reaching approximately 90% confluence (typically after two to three days), viability being assessed by Trypan Blue exclusion; cells were then detached with 0.25% trypsin and subcultured at a 1:3 split ratio.

One of the 3 vials of cells was used to characterize the cells. This was performed by flow cytometry, following the presence of specific markers for stem cells: CD29, CD44, CD73, CD90, and CD117, concomitant with the absence of specific markers for hematopoietic cells: CD14, CD31, and CD105.

### 2.3. Isolation, Characterization, and Transfection of EVs

Stem cells isolated from the spinal cord and adipose tissue were kept in culture until passage 5, following a period of 48 h of their incubation in a serum-free medium to release the EVs (BMMSCs) or EVs (ADSCs)). The next stage involved a series of centrifugations and ultracentrifugations to obtain EVs (microvesicles (MVs) or microparticles (MPs) and exosomes), as described by Comaritia et al., 2022 [35]. More precisely, in the first step, we followed the removal of cellular debris by centrifugation at 2500× *g* for 10 min, while the second centrifugation step followed the elimination of apoptotic bodies (16,000× *g*, 5 min). Next, the supernatant was taken and 2 ultracentrifugation steps were performed as follows: (1) at 100,000× *g*, 20 h, at 4 °C—to obtain EVs in pellet; (2) at 100,000× *g*, 2 h 30′, at 4 °C—to get purified EVs after washing them with sterile PBS. Since the pellet contains both exosomes and MVs, their characterization was required. Considering the size differences between exosomes (50 nm–100 nm) and MVs (100 nm–1000 nm), the size of the vesicles was determined using Malvern Zetasizer Nano ZS. Later, the vesicles were characterized by flow cytometry, monitoring the presence of annexin V—a marker specific to MVs, respectively, of CD63—an exosomal marker. We also performed additional checks using the technique of transmission electron microscopy (TEM). This assumed incubation of a sample with 5 μL EVs on grids at room temperature for 2 min, followed by staining with 2% uranyl acetate, and, finally, images were captured using a 200 kV Talos F200C TEM (ThermoFisher Scientific, Waltham, MA, USA).

Next, we focused on the transfection of EVs (EVs (BMMSCs) or EVs (ADSCs)) with Smad2/3 siRNA (100 nM final concentration) (Santa Cruz Biotechnology, Inc., Santa Cruz, CA, USA) carried out in accordance with the manufacturer’s protocol. For this, we used 2 specific compounds intended to ensure the efficiency of the transfection, respectively, Lipofectamine™ RNAiMAX transfection reagent (Thermo Fisher Scientific, Waltham, MA, USA) and OPTI-MEM (Gibco by Life Technologies, Waltham, MA, USA), an optimized medium that reduces cell damage that can occur during transfection. Using these, we made two mixtures: one with SMAD 2/3 siRNA (3 µL) and OPTI-MEM (50 µL) and the other one with Lipofectamine (3 µL) and OPTI-MEM (50 µL). After the homogenization of these two mixtures, the EVs (100 μg/mL) were added and left to incubate and perform easy homogenization for 3 days at 37 °C in 5% CO_2_ atmosphere, and then they were ultracentrifuged at 100,000× *g* for 2 h at 4 °C. Finally, the supernatant was removed and the pellet containing the transfected EVs was resuspended with 300 µL PBS. In parallel, EVs were transfected with siRNA-FITC as a positive control, and the transfection efficiency was verified by flow cytometry [35].

### 2.4. Characterization of the Group Animals

The effects of the atherogenic diet (high in 3% cholesterol and 15% butter), gavage (with 8% NaCl), and EV treatment were evaluated during a 4-month experimental period. Thus, at the beginning of the experiment, and then every month, the animals were weighed and venous blood from the retro-orbital plexus was collected. This was conducted under anesthesia by inhalation of 2% isoflurane. The blood was centrifuged (2500× *g*, 10 min) to obtain plasma, after which the plasma parameters were evaluated as follows: cholesterol, HDL, LDL, triglycerides, and glucose, using DIALAB GmbH (Vienna, Austria) kits based on the colorimetric principle, obtaining a color reaction in which the intensity is directly proportional to the concentration. Thus, spectrophotometric measurements were made at 500–600 nm using the Tecan Infinite M200 Pro (Tecan Group Ltd., Männedorf, Switzerland) spectrophotometer.

### 2.5. Evaluation of Cardiac Hypertrophy by Echocardiography

In order to investigate cardiac hypertrophy in hamsters from the experimental groups, the Vevo 2100 equipment (VisualSonics Inc., Toronto, ON, Canada) was used, as described in the paper published by Alexandru et al., 2020 [25]. During the analysis, the hamsters were anesthetized with 2% isoflurane, with body temperature and heart rate constantly monitored. The animals were placed on a special table in a parasternal position, and the chest wall was palpated in the lower part, more precisely between the IV and VII pair of ribs, a transductor being applied at the level of the apexian shock. The apexian shock is a cardiac impulse typically produced by the left ventricle, providing essential information about the presence of various cardiac pathologies. After placing the transducer, translational and rotational movements were made so that the tip remained fixed to obtain clear and relevant images.

The blood flow through the aortic valve, also known as transvalvular velocity and the velocity–time integral at the level of the ejection tract of the left ventricle through the pulsed Doppler module, was evaluated. The velocity–time integral is used to determine the cardiac output, as well as the systolic function of the left ventricle. In addition, a B-mode echocardiographic examination was also performed to allow an analysis of the thickness of the anterior and posterior wall of the left ventricle and the size of the ventricular cavity as well as wall movements. Later, on top of the B-mode, the M-mode was activated which allowed us to obtain some graphics on movement time. Thereby, we were able to determine the cardiac output, shortening fraction, and ejection fraction. The shortening fraction was calculated by subtracting the systolic diameter from the diastolic diameter of the left ventricle, while the ejection fraction is the ratio between the stroke volume and end-diastolic volume. Both parameters are useful for assessing the systolic function of the left ventricle. The images thus obtained were later analyzed using the VevoLab300 software, version 5.9.0 (FUJIFILM VisualSonics, Toronto, ON, Canada).

### 2.6. Investigating the Key Regulators, Ang II and TGF-β1, in the Atherosclerotic Process and the Associated Cardiac Hypertrophy by ELISA Technique

TGF-β1 and AngII were quantified in plasma in order to compare their levels across the seven experimental hamster groups. More precisely, the plasma levels of these markers were determined, using the plasma obtained by centrifugation at the end of the 4 months of diet and gavage, and commercial kits: Human/Mouse TGF-beta 1 Uncoated ELISA Kit (Invitrogen, Carlsbad, CA, USA) and Angiotensin II EIA Kit (Sigma-Aldrich, Saint Louis, MO, USA). All the stages provided in the kit protocol were completed, namely, adding the capture antibody, the blocking solution, and preparation of the samples, as well as the necessary solutions for the standard curves, and addition of the detection antibody. These were pipetted in triplicate for the accuracy of the results. In the end, the substrate—TMB (3, 3′, 5, 5′ Tetramethylbenzidine)—was added. TMB is a substrate-photosensitive chromogen that acts as a hydrogen donor for peroxide reduction of hydrogen to water in the presence of peroxidase (HRP). As a result of the reaction, the mix acquires a blue color that can be measured with a spectrophotometer. The reaction is stopped with a stop solution represented by sulfuric acid which causes the blue color to change to yellow. The last step involves reading the plates with the spectrophotometer (Tecan Infinite M200 PRO, Tecan Group Ltd., Männedorf, Switzerland) at the 450 nm wavelength.

### 2.7. In Situ Detection of Reactive Oxygen Species Generation in the Left Ventricle

The level of reactive oxygen species produced in the left ventricle was able to be analyzed and subsequently quantified by immunofluorescence. The principle of the method consists of the use of fluorochrome dihydroethidium (DHE). The DHE in the presence of superoxide is rapidly oxidized to ethidium bromide (EtBr), which is a mutagenic agent with the ability to intercalate between the nitrogenous bases of DNA, being often used for visualization of the nucleic acid fragments in electrophoresis. This property of the EtBr is the basis of the method, the intercalation of EtBr in the DNA structure leading to the appearance of a red fluorescence visible under the fluorescence microscope [36]. Basically, over the blades showing left ventricular sections from the seven groups of hamsters, 6 μM DHE was added and incubated at room temperature in the dark for 40 min. The slides were subsequently analyzed under a fluorescence microscope (Axio Vert. A1 Fl, Carl Zeiss, AxioVision Rel 483SE64-SP1software, 20× objective). The images were quantified using ImageJ software, version 1.54.

### 2.8. Analysis of Specific Inflammatory Markers with Roles in Atherosclerosis and in Associated Cardiac Hypertrophy by Immunohistochemistry

At the end of the 4 months of diet and treatment, the hamsters were perfused with PBS and 1 mM CaCl_2_ in order to collect the organs of interest (the left ventricle) for structural analyses by immunohistochemistry. After cutting the ventricle into pieces, they were suspended in a 2% paraformaldehyde solution (PFA) and left overnight. Later, they carried out successive baths with glycerol for tissue cryoprotection, as follows: 5% glycerol 15 min at room temperature, 10%, 1 h at 4 °C and 20% overnight at 4 °C. The next day 50% glycerol was added for one hour at 4 °C. Afterward, the glycerol bath was changed to fresh of the same concentration in which the tissue was stored at −20 °C until preparation techniques.

When tissue sectioning was desired, the samples were reacclimatized to 4 °C, followed by three PBS washes and six 3% sucrose baths for 15 min, sucrose having a cryoprotective role. The next step consisted of including the fragments of the left ventricle in the OCT (Optimal Cutting Temperature) for 30 min at room temperature, followed by freezing fast with liquid nitrogen. The thus manipulated tissue was mounted on a special support with OCT and left in cryotome (Leica, Teaneck, NJ, USA) overnight. Later, tissue sections were obtained at the cryotome, which were attached to special slides coated with poly-L-lysine that allows optimal adhesion of tissue. The thickness of the sections obtained was 5 μm, a thickness considered optimal for analyzing the proteins of interest, as thicker sections have a disadvantage interpretation of the fluorescent signal because they present several focal planes.

The slides thus obtained were washed with PBS buffer and fixed for 10 min at −20 °C in methanol. Methanol is a chemical fixative that has the role of precipitating proteins’ surface. Subsequently, the slides were immersed in a solution of 1 mg/mL sodium borohydride (NaBH_4_) for one hour at 4 °C. This has the role of eliminating possible aldehyde groups [37] remaining after the PFA fixation step, but also to prevent the appearance of autofluorescence. Next, the slides were washed three times with PBS and subsequently immersed in a solution of 0.2% Triton X-100/0.05% Tween 20 in PBS for 30 min at room temperature. Afterwards, we repeated the washes steps with PBS, followed by the blocking phase with 10% goat serum for 10 min. Then followed the incubation with the first antibody corresponding to the protein of interest, diluted in a PBS solution with 1% BSA. The proteins investigated were connexin 43 (1:200, Thermo Fisher Scientific), cardiac troponin I (1:1000. Thermo Fisher Scientific), macrophage migration inhibitory factor-MIF (1:200, Santa Cruz Biotechnology), atrial natriuretic factor-ANF (1:200, Santa Cruz Biotechnology), collagen type I (1:250, Santa Cruz Biotechnology), α-SMA (1:200, Cell Signalling Technology), CD206 (1:200, Santa Cruz Biotechnology), MHC-II (1:250, Thermo Fisher Scientific), CD68 (1:200, Santa Cruz Biotechnology), and CD3e (1:200, ThermoFisher Scientific). After incubation with the primary antibody, the slides were washed and the corresponding secondary antibody, anti-species in which the primary antibody is produced and coupled with a fluorophore, was added (Alexa 488 goat anti-rabbit IgG (H + L) 1:500 (green), Alexa 568 goat anti-rabbit IgG (H + L) 1:1000 (red), and Alexa 647 donkey anti-mouse IgG (H + L) 1:500 (Red)). After incubation with the secondary antibody, 3 washes with PBS were performed, followed by incubation with DAPI (4′, 6-diamidino-2-phenylindole) (5 mg/mL in PBS—10 mM). DAPI is a fluorescent dye that specifically binds to adenine–thymine-rich regions of DNA, thus highlighting the nuclei in the tissue [38]. After incubation with DAPI for 5 min, the slides were washed, and a mounting fluid (ProLong, Invitrogen) was added to polymerize and fix the slide.

The slides thus processed were visualized under a phase-contrast microscope (Axio Vert.A1 Fl, Carl Zeiss, AxioVision Rel 483SE64-SP1 software) at 20× objective, and the obtained images were analyzed using ImageJ software.

### 2.9. Exploring the Mechanisms by Which Diet- and Gavage-Induced Cardiac Hypertrophy Signaling Pathways Are Affected

The left ventricle fragments taken from the seven experimental groups of hamsters were cut into small pieces and homogenized using an ultrasonic homogenizer. Afterwards, the samples were centrifuged, the supernatant being the one of interest. Finally, the protein concentration was measured using the BCA method (Bicinchoninic Acid Assay).

Then, a double denaturation was performed using beta-mercaptoethanol and heating at 100 °C for 5 min. Subsequently, proteins (100 μg/lane) were separated by migration in 10% polyacrylamide denaturing gel (SDS-PAGE). A wide-range molecular weight marker (6.5 ÷ 200 kDa) (Sigma) was loaded into one lane as a standard. The protein bands of the obtained gel were transferred with the help of two electrodes on nitrocellulose paper in a semi-dry system. After making the transfer, the blocking stage follows, the purpose of which is to prevent the binding of non-specific antibodies to the hydrophobic sites left free. Two blocking solutions were used, respectively, for the detection of phosphorylated forms of proteins of interest, and the blocking was performed with a solution of 3% bovine serum albumin (BSA) in PBS supplemented with 0.05% Tween 20, while for the detection of unphosphorylated forms, blocking was conducted with a 5% milk solution in PBS with 0.05% Tween 20, for 1 h at room temperature. Next, the membranes were washed with PBS with 0.05% Tween 20, and then they were incubated with the appropriately diluted primary antibody specific to each protein of interest, respectively, mouse monoclonal antibodies: SMAD2 (1:1000, Thermo Fisher Scientific), and rabbit polyclonal antibodies: GAPDH (1:1000, Thermo Fisher Scientific), NF-kB p50 (1:200, Santa Cruz Biotechnology). Incubation was performed overnight at 4 °C. The following day, the membranes were washed and incubated with the appropriate secondary antibody, an anti-species antibody in which the first antibody is produced (anti-mouse Ac 1:5000, Thermo Fisher Scientific, anti-rabbit Ac1:2000, Thermo Fisher Scientific) for 1 h at room temperature. Secondary antibodies were coupled with HRP (Horse Radish Peroxidase), the detection of the bands being chemiluminescent. Specifically, conjugated HRP with the secondary antibody cleaved the HRP substrate (luminol + peroxide) resulting in a luminescent product whose intensity is proportional to the amount of protein present. The light generated as a result of this reaction was detected with the help of a film pressed on the membrane which was later developed using the ECL detection system (Enhanced Chemiluminescence).

The images thus obtained were analyzed by densitometry using the TotalLab’s analysis software. To quantify the amount of protein (protein expression), the samples of interest corresponding to the investigated experimental groups were reported to the band corresponding to GAPDH (Glyceraldehyde 3-Phosphate Dehydrogenase), protein that is constitutively expressed in the mammalian heart.

## 3. Results

### 3.1. Characterization of ADSCs, BMMSCs, and EVs Derived from Them

The data obtained using flow cytometry demonstrated that both ADSCs and BMMSCs were positive for several well-established stem cell markers, including CD29, CD44, CD73, CD90, CD105, and CD117, while they were negative for hematopoietic cell markers such as CD14, CD31, and CD45.

The results derived from the analysis of the size, structure, distribution, and composition of EVs have been previously published in an article by Comarita and Vilcu (2022) [35].

### 3.2. Impact of Atherogenic Diet and Treatments on Key Parameters

The effects of an atherogenic diet and therapeutic interventions were studied in seven experimental groups of hamsters: control (C), HH (hyperlipidemic–hypertensive), HH treated with EVs derived from ADSCs (HH-EVs (ADSCs)), HH treated with EVs derived from BMMSCs (HH-EVs (BMMSCs)), HH treated with EVs derived from ADSCs transfected with Smad2/3 siRNA (HH-EVs (ADSCs) + Smad2/3siRNA), HH treated with EVs derived from BMMSCs transfected with Smad2/3 siRNA (HH-EVs (BMMSCs) + Smad2/3siRNA), and HH group treated with Smad2/3 siRNA alone (HH-Smad2/3siRNA). These groups were assessed for body weight and plasma parameters, the lipid profile and glucose, at baseline and monthly over a four-month period. Throughout this period, all HH groups were fed an atherogenic diet (15% butter and 3% cholesterol) and received daily gavage of 8% NaCl, while the control group was fed with standard chow.

#### 3.2.1. Body Weight Trends

**Figure 1:**
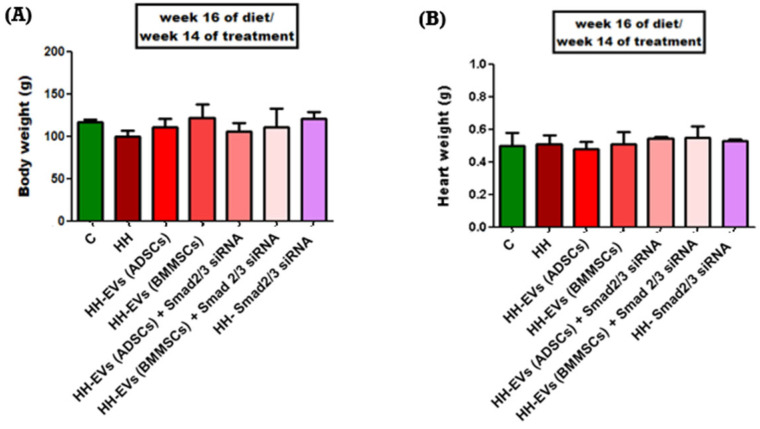
Analysis of body (**A**) and heart weights (**B**) collected at the end of the diet and treatment period for the seven experimental groups. Values are expressed as mean ± standard deviation (SD). The values were calculated by two-way ANOVA, Bonferroni post-test.

The hamsters in the HH group showed a slight but statistically insignificant decrease in weight after two, three, and four months of the diet. In contrast, the weight of the control group remained stable throughout the experiment. The treated groups, particularly HH-Smad2/3siRNA and HH-EVs (BMMSCs), exhibited small increases in weight (Figure 1A). Hearts were collected and weighed at the end of the experiment. No significant differences in heart weight were observed among the seven experimental groups (Figure 1B).

#### 3.2.2. Plasma Biochemical Changes

Compared to the control group, HH animals showed significant increases in plasma levels of total cholesterol, LDL cholesterol, and triglycerides over the experimental period. HDL cholesterol levels in the HH group remained unchanged during the first two months but increased significantly during the fourth month. Blood glucose levels remained stable throughout the experiment in all groups (Table 1).

The therapeutic approach resulted in significant improvements in plasma lipid profiles, with EVs derived from ADSCs and BMMSCs effectively reducing total cholesterol, triglycerides, and LDL cholesterol. The combined administration of EVs and Smad2/3 siRNA further reduced lipid levels, with effects comparable to those observed with EV treatment alone. In contrast, the HH-Smad2/3siRNA group exhibited a significant increase in triglyceride levels compared to both the control and treated groups (Table 1).

The seven groups included control (C), hypertensive–hyperlipidemic (HH), HH-EVs (ADSCs), HH-EVs (BMMSCs), HH-EVs (ADSCs) + Smad2/3siRNA, HH-EVs (BMMSCs) + Smad2/3siRNA, and HH-Smad2/3siRNA. Animals were subjected to either a standard diet or an atherogenic diet, followed by treatments with extracellular vesicles (EVs) derived from ADSCs or BMMSCs, transfected or not with Smad2/3siRNA.

#### 3.2.3. Observations on Liver and Blood

**Figure 2:**
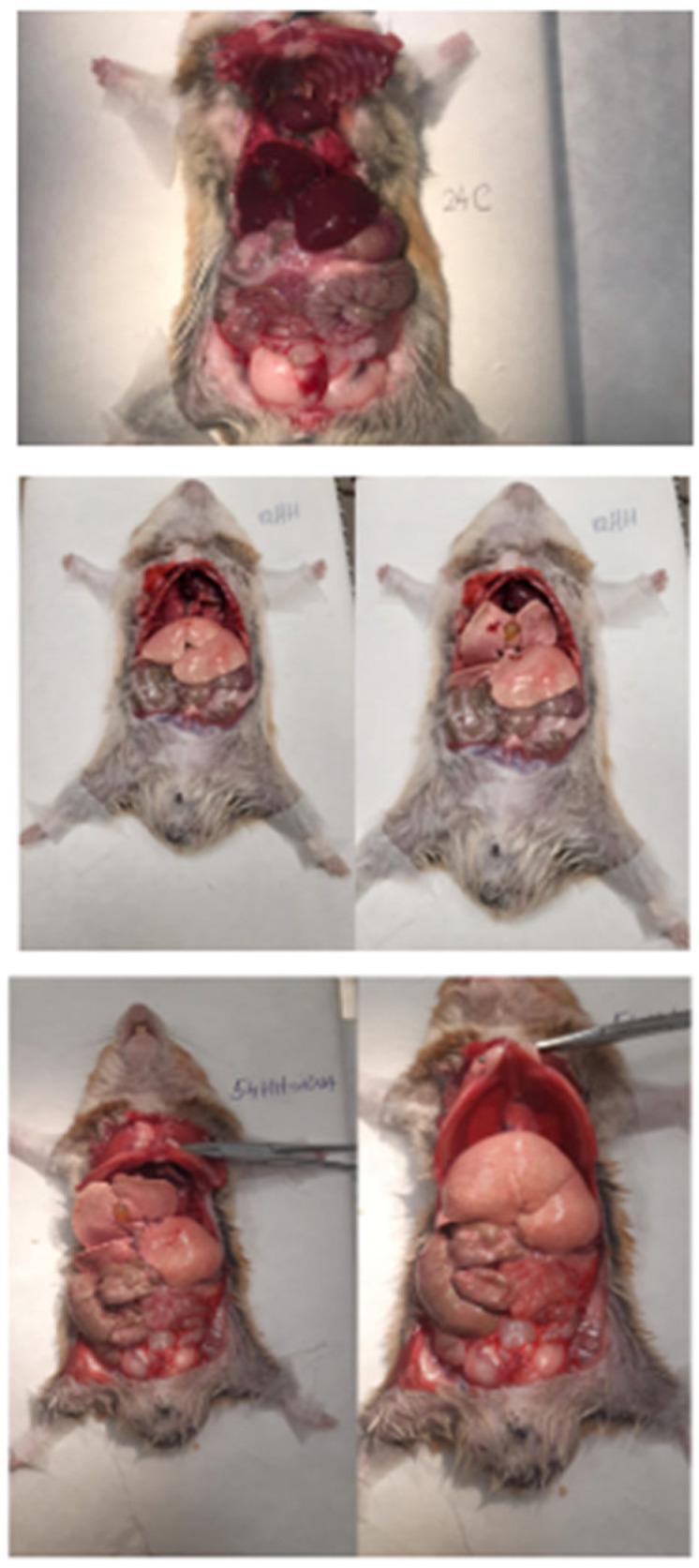
Representative anatomical observations of the abdominal and thoracic cavities in experimental groups of animals subjected to different dietary and therapeutic conditions. The first panel (24C) illustrates the control group with normal liver morphology and organ appearance, indicative of a standard physiological state. The second row (81HH) displays a subject from the hypertensive–hyperlipidemic (HH) group, with pathological changes including an enlarged, yellowish, and fatty liver consistent with hepatic steatosis, together with a more congested appearance of the visceral tissues. In the third row, a subject from one of the treated groups (54HH-Smad2/3 siRNA) shows visible improvements in liver morphology, with a reduction in the yellowish discoloration and fatty texture. The overall appearance of the abdominal and thoracic organs in the treated animals appears to be more consistent with that of the control group, indicating a therapeutic effect of the administered treatment.

The atherogenic diet resulted in noticeable liver damage, characterized by a yellow, fatty appearance indicative of hepatic steatosis (Figure 2), and plasma with a milky consistency due to elevated lipid concentrations. In contrast, the treated groups showed improved liver morphology and plasma clarity, suggesting a significant reduction in lipid accumulation (Figure 2).

#### 3.2.4. Confirmation of the Animal Experimental Model of Human Atherosclerosis

The changes in the lipid profile in the HH group, together with previous findings of elevated systolic and diastolic blood pressure and heart rate [21] confirmed the development of a hypertensive–hyperlipidemic model. This model effectively mimics human risk factors for atherosclerosis, supporting the validity of the experimental design.

### 3.3. Assessment of Alterations at the Structural and Functional Levels in the Left Ventricle as a Key Part of the Atherosclerotic Process

**Figure 3:**
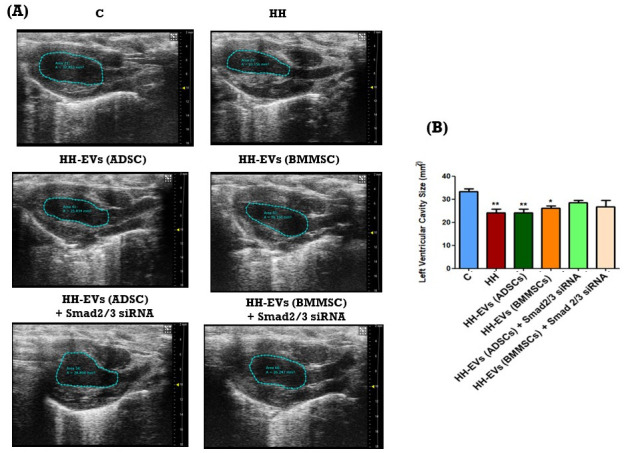

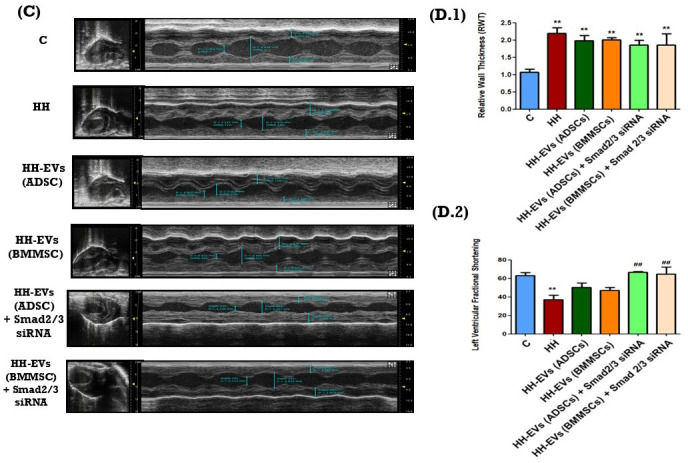
Left ventricular structural and functional parameters assessed across experimental groups following dietary and therapeutic interventions. Left ventricular cavity size (mm^2^) (**B**), relative wall thickness (RWT) (**D.1**), and left ventricular fractional shortening (%) (**D.2**) were evaluated to assess cardiac remodeling and function. (**A**) Left ventricle—internal cavity—representative recordings, long axis, in B-mode; (**B**) graphical representation of internal cavity (mm^2^); (**C**) left ventricle—diameter in diastole and systole/anterior and posterior wall thickness—representative recordings in M-mode; (**D.1**) graphical representation of relative wall thickness; (**D.2**) graphical representation of left ventricular shortening fraction. The data presented herein are expressed as means ± standard deviation (SD), based on duplicate measurements. Statistical significance is indicated as follows: ** *p* < 0.01, and * *p* < 0.05 compared to the control group; **^##^** *p* < 0.01 compared to the HH group. Two-way ANOVA, Bonferroni post-test was applied.

Cardiac hypertrophy occurs when cardiomyocytes increase in size, leading to impaired contractile function. Pathological cardiac hypertrophy is primarily induced by hypertension and the mechanical stress exerted on the myocardium [14]. However, its development is also influenced by comorbidities such as obesity, diabetes mellitus, and atherosclerosis [39]. Specifically, heightened blood flow pressure has been demonstrated to stimulate the secretion of growth factors and cytokines, which in turn promote myocardial mass enlargement. To assess the onset of cardiac hypertrophy, particularly of the left ventricle, echocardiography, a widely used technique in the diagnosis of cardiovascular disease, has been employed. Both the structure and function of the left ventricle were examined and key parameters such as internal cavity size, diastolic and systolic diameters, and anterior and posterior wall thickness were analyzed (Figure 3). These measurements were made using both B-mode and M-mode imaging, providing a comprehensive and detailed assessment of ventricular morphology and function.

In comparison with the control group, the HH hamsters demonstrated a substantial decrease in left ventricular cavity size (approximately 30%), accompanied by significant thickening of the ventricular wall (** *p* < 0.01). These findings indicate that a diet comprising butter and cholesterol, in conjunction with NaCl gavage, resulted in the onset of cardiac hypertrophy (Figure 3A,B). In the groups of hamsters receiving both the atherogenic diet, gavage, and treatment with non-transfected EVs, a significant reduction in the size of the left ventricular cavity was observed. In contrast, the groups treated with Smad2/3 siRNA-transfected EVs showed a slight improvement in cavity size, but this improvement was not statistically significant (Figure 3B). The measurements were obtained by means of transthoracic echocardiography using the B-mode.

Utilizing M-mode recordings of diastolic and systolic diameters, in conjunction with the anterior and posterior wall thickness of the left ventricle (Figure 3C), two additional parameters were quantitatively analyzed to assess structural and functional changes: the relative wall thickness and the left ventricular shortening fraction. The results demonstrated that in all hamsters subjected to daily gavage and the atherogenic diet, the relative wall thickness of the left ventricle was significantly increased compared to the control group (** *p* < 0.01) (Figure 3(D.1)).

In what concerns the function of the left ventricle assessed by determination of the shortening fraction, it was significantly reduced in the HH group (** *p* < 0.1). As far as that goes, for treated groups, significant improvements (**^##^** *p* < 0.01) were observed in the two groups treated with Smad2/3 siRNA-transfected EVs (HH-EVs (ADSCs) + Smad2/3 siRNA and HH-EVs (BMMSCs) + Smad2/3 siRNA (Figure 3(D.2)).

Although no substantial structural enhancements were observed in the treated groups, this observation does not extend to functional parameters. It is noteworthy that left ventricular elasticity was enhanced in the treated groups, particularly in those who received transfected EVs, underscoring the therapeutic potential of these particles.

### 3.4. Investigation of Key Regulators in the Atherosclerotic Process and Associated Cardiac Hypertrophy with Focus on TGF-β1 and Ang II

Transforming growth factor-beta 1 (TGF-β1) has been identified as a critical regulator in various cellular processes, including growth, differentiation, apoptosis, and inflammation [40]. Elevated expression of TGF-β1 has been closely associated with cardiac hypertrophy, cardiomyocyte apoptosis, and cardiac fibrosis [41]. Notably, individuals afflicted with atherosclerosis exhibit elevated TGF-β1 levels, a phenomenon attributed to the influx of macrophages into atherosclerotic lesions, which secrete elevated levels of TGF-β1 and Angiotensin II (AngII). This excessive production of bioactive molecules has been observed to stimulate uncontrolled extracellular matrix synthesis, thereby exacerbating the progression of pathological processes [42].

AngII, a pivotal element of the renin–angiotensin–aldosterone system, demonstrates vasoconstrictive characteristics, resulting in elevated blood pressure and platelet aggregation. Moreover, AngII contributes to the augmentation of cardiac cells by means of activating protein kinase C, thereby facilitating the initiation of cardiac hypertrophy [43].

In order to assess the involvement of these markers, the concentrations of TGF-β1 and AngII in the plasma of hamsters from the seven experimental groups were analyzed using the ELISA technique. More specifically, at the conclusion of the four-month diet and gavage regimen, the concentrations of these markers were quantified and normalized to the corresponding standard curves.

**Figure 4:**
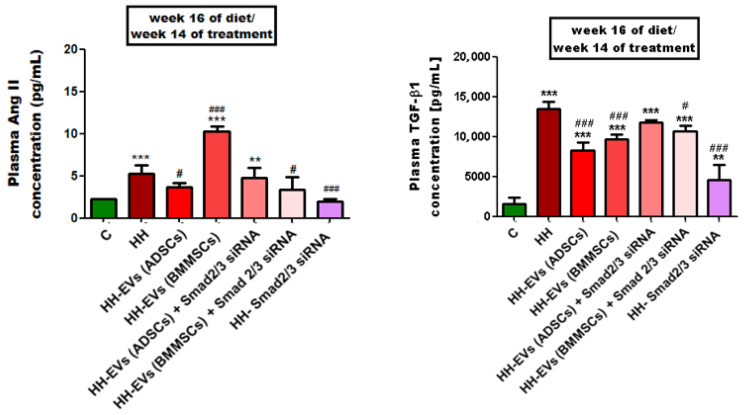
ELISA quantification of plasma levels for TGF-β1 and AngII for the seven experimental groups is presented as means ± standard deviation (SD) based on triplicate measurements. Statistical significance is indicated as follows: ** *p* < 0.01, *** *p* < 0.005 compared to the control group; **^#^** *p* < 0.05, and **^###^** *p* < 0.005 compared to the HH group. Two-way ANOVA, Bonferroni post-test was applied.

The results demonstrated that the HH group exhibited a significant increase (*** *p* < 0.005) in plasma concentration of both markers (Ang II and TGF-β1) in comparison to the control group. Conversely, significant improvements were observed in the most treated groups. Regarding plasma AngII levels, substantial reductions were observed in the HH-EVs (ADSCs), HH-EVs (BMMSCs) + Smad2/3 siRNA, and HH-Smad2/3 siRNA groups (Figure 4).

In the case of TGF-β1, it was determined that the administration of treatment lead to a significant decrease (**^#^** *p* < 0.1, **^###^** *p* < 0.005) in plasma concentrations when compared to the HH group, with the most noticeable improvements for the following groups: HH-EVs (ADSCs), HH-EVs (BMMSC), HH-EVs (BMMSCs) + Smad2/3 siRNA, and HH-Smad2/3 siRNA (Figure 4).

### 3.5. Measurement of Reactive Oxygen Species in the Left Ventricle

Reactive oxygen species (ROS) have been demonstrated to play a crucial role in cardiovascular physiology, contributing to the initiation, progression, and exacerbation of associated diseases. Several risk factors associated with atherosclerosis and hypertrophy, including smoking, hypercholesterolemia, and environmental pollution, have been shown to promote increased levels of ROS in the myocardium and vascular wall [44]. When ROS production exceeds the protective capacity of antioxidant defense systems, a cascade of pathological events is triggered, ultimately leading to atherosclerotic plaque formation. These events include endothelial dysfunction, LDL oxidation, leukocyte migration, and extracellular matrix degradation [45].

**Figure 5:**
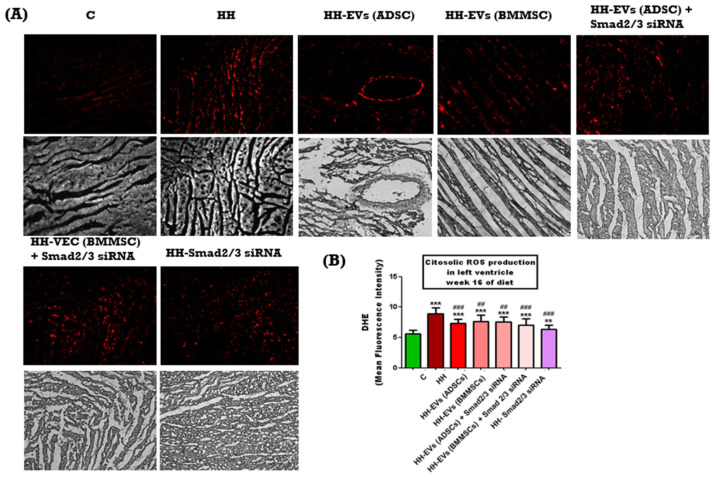
Assessment of reactive oxygen species production (ROS) in the left ventricle of the seven experimental groups at the end of 4 months of diet and/or treatment. (**A**) Representative fluorescence and phase-contrast images taken under an inverted microscope (20× magnification) to highlight the level of reactive oxygen species in the left ventricle of the C, HH, HH-EVs (ADSCs), HH-EVs (BMMSCs), HH-EVs (ADSCs) + Smad2/3 siRNA, HH-EVs (BMMSCs) + Smad2/3 siRNA, HH-Smad2/3 siRNA. (**B**) Graphical representation of the levels of reactive oxygen species in the left ventricle. Data are presented as mean ± standard deviation (SD) based on duplicate measurements. Statistical significance is indicated as *** *p* < 0.005, ** *p* < 0.01 compared to the control group; **^###^** *p* < 0.005, **^##^** *p* < 0.01, compared to the HH group. Two-way ANOVA, Bonferroni post-test was applied.

In order to assess the elevated ROS levels in the left ventricle of hamsters from the seven experimental groups, an immunohistochemical technique was employed, using a special agent dihydroethidium (DHE), a fluorescent probe that, upon oxidation by ROS, is converted into ethidium bromide, emitting red fluorescence. Phase-contrast microscopy imaging, followed by quantitative analysis using ImageJ software, revealed a significantly increased ROS level in the HH group compared to the control group (*** *p* < 0.005) (Figure 5B). This increase was found to be strongly correlated with left ventricular hypertrophy and the presence of atherosclerotic lesions (Figure 5A). Conversely, all treated groups exhibited significantly lower ROS levels compared to the HH group (^###^ *p* < 0.005, ^##^ *p* < 0.01) (Figure 5B) suggesting that the administered treatment may effectively interrupt the cascade of events leading to atherosclerotic plaque formation and cardiac hypertrophy.

### 3.6. Analysis of Inflammatory Markers Involved in Atherosclerosis and Associated Cardiac Hypertrophy

**Figure 6:**
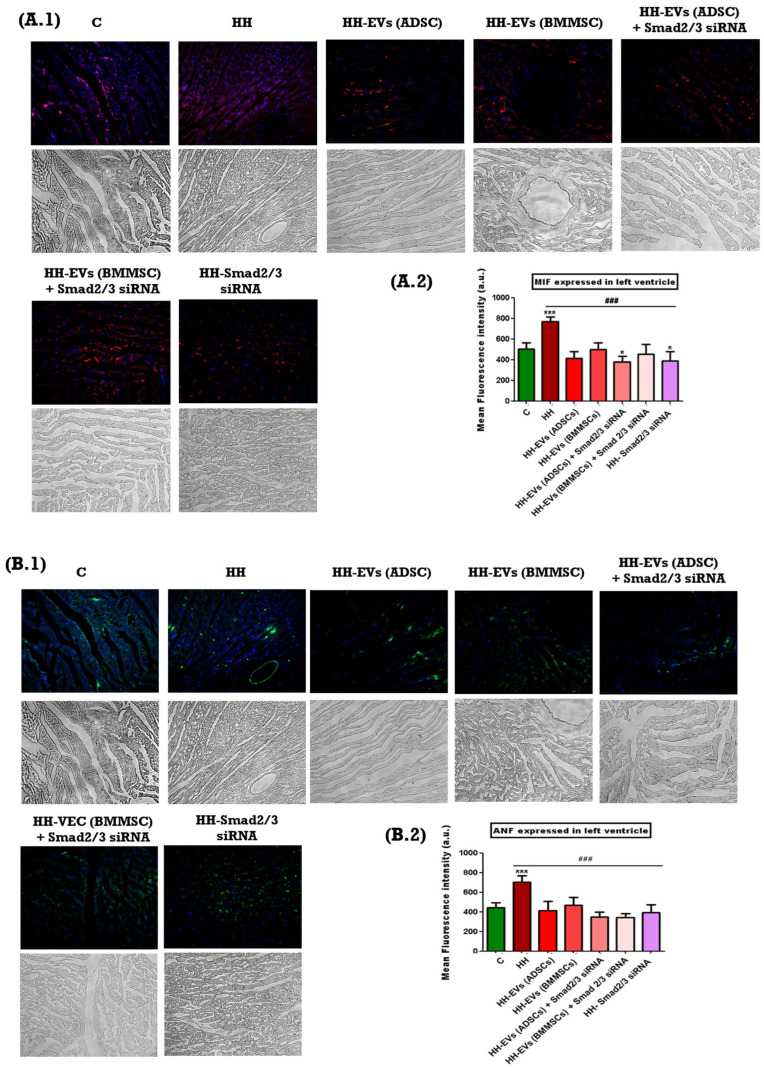

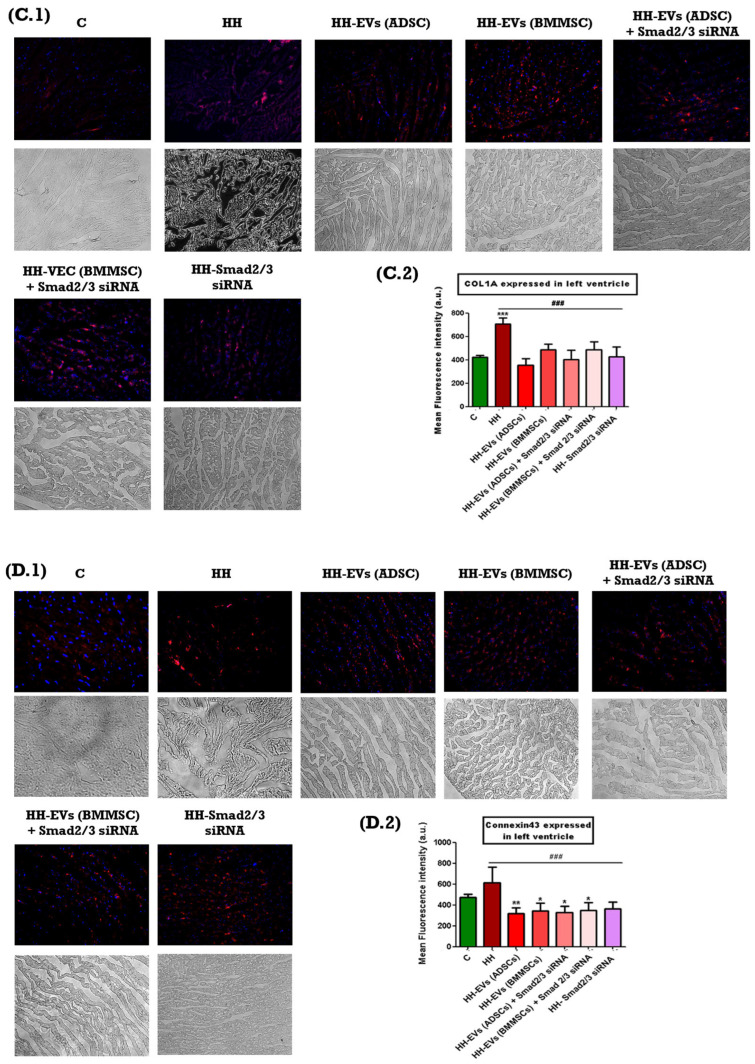

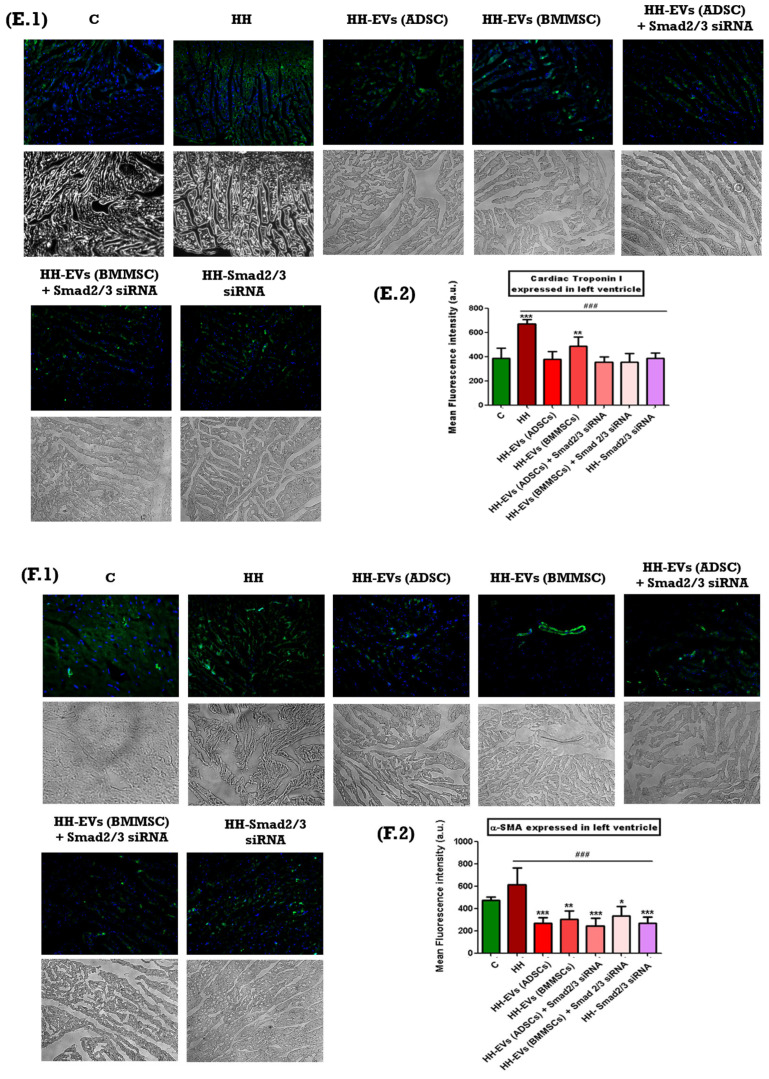

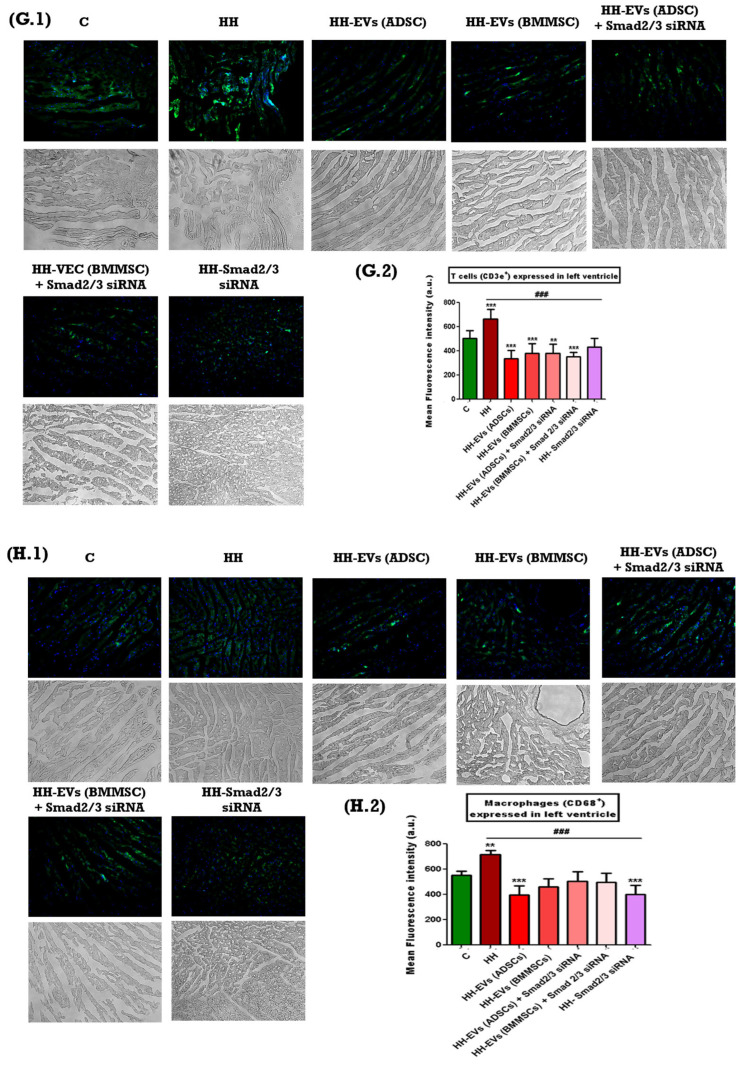

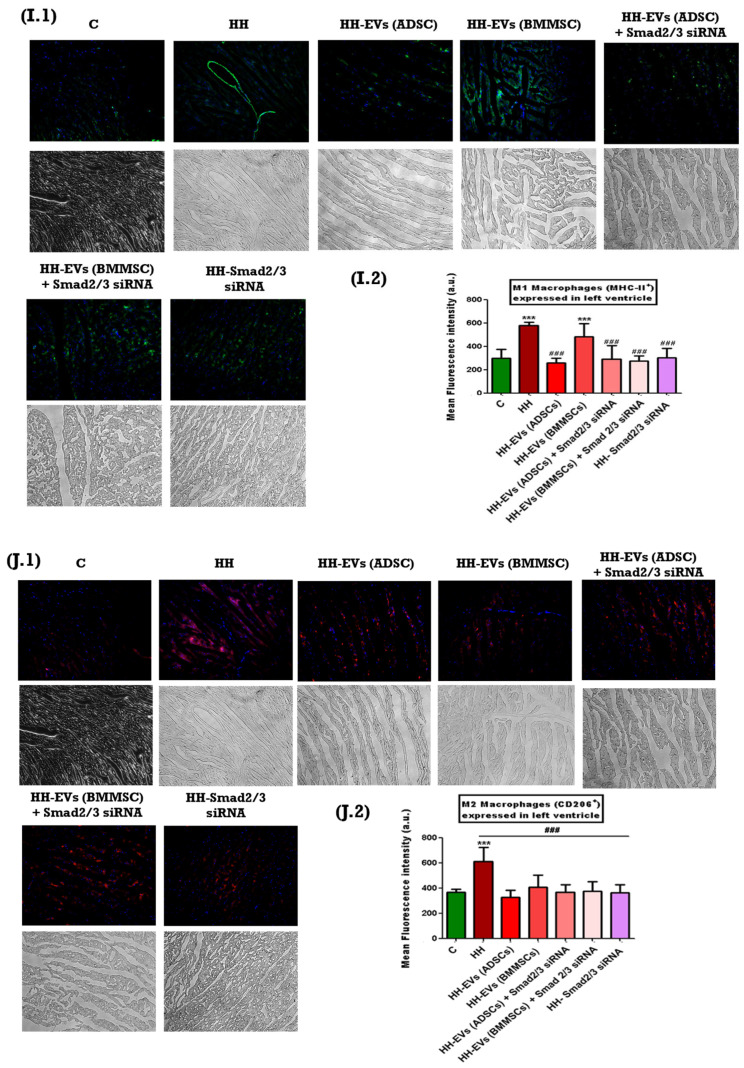
Illustrative immunofluorescence images showing inflammatory markers and cell types associated with cardiac hypertrophy and atherosclerosis at the end of the diet and/or treatment. Thin cryosections of the left ventricle collected from the seven experimental groups were labeled as follows: (**A.1**) MIF, (**B.1**) ANF, (**C.1**) COL1A, (**D.1**) Cx43, (**E.1**) CTN1, (**F.1**) α-SMA, (**G.1**) T cells, (**H.1**) macrophages, (**I.1**) M1 macrophages, and (**J.2**) M2 macrophages. Nuclei were visualized by DAPI staining in blue fluorescence. Images were taken at a total magnification of 20×. (**A.2**–**J.2**)—graphical representation of the levels of the assessed markers in the left ventricle. Data are presented as mean ± standard deviation (SD) based on triplicate measurements. Statistical significance is indicated as *** *p* < 0.005, ** *p* < 0.01, and * *p* < 0.05 compared to the control group; **^###^** *p* < 0.005, compared to the HH group. Two-way ANOVA, Bonferroni post-test was applied.

The presence of specific inflammatory markers associated with cardiac hypertrophy mentioned previously was investigated through immunofluorescence on left ventricular tissue sections from the seven experimental groups (C, HH, HH-EVs (ADSCs), HH-EVs (BMMSCs), HH-EVs (ADSCs) + Smad2/3 siRNA, HH-EVs (BMMSCs) + Smad2/3 siRNA, HH-Smad2/3 siRNA). Phase-contrast microscopy (20× magnification) followed by quantitative analysis using ImageJ software revealed a significant increase (*** *p* < 0.005, ** *p* < 0.01) in the infiltration of the previously mentioned inflammatory markers in the HH group compared to the control group (Figure 6). For the treated groups, significant reductions (**^###^** *p* < 0.005) were observed in all investigated markers, with the exception of M1 macrophages. For this particular marker, enhancements were also evident in all treated groups, apart from the group that received EVs obtained from bone marrow stem cells (HH-EVs (BMMSCs) group) (Figure 6).

### 3.7. The Role of TGF-Β1 Signaling Pathways in Atherosclerosis-Associated Cardiac Hypertrophy

A comprehensive analysis of the key signaling pathways involved in atherosclerosis-associated cardiac hypertrophy was considered necessary to facilitate a complete understanding of this pathology and to evaluate the therapeutic potential of EVs, both transfected and non-transfected with Smad2/3 siRNA.

The pathogenesis of atherosclerosis is associated with two major signaling pathways: TGF-β1/Smad2/3 and TGFβ1/TAK1/Nf-κB. Upon binding to its specific receptors, TGF-β1 triggers the activation of Smad2/3, forming a complex that translocates to the nucleus, where it promotes the transcription of genes involved in the synthesis of the extracellular matrix (ECM) and cardiac fibrosis [46]. The overexpression of these genes has been found to be closely associated with cardiac hypertrophy, pathological remodeling, and the formation and potential rupture of fibro-atheromatous plaques.

Concurrently, the non-canonical TGF-β1/TAK1 pathway activates key molecules, including Nf-κB and AP-1. These molecules, in turn, enhance the expression of genes encoding adhesion molecules (ICAM-1 and VCAM-1), selectins, and matrix metalloproteinases [47]. The combined effect of these molecules is to contribute to vascular inflammation and plaque instability.

In order to further investigate these molecular mechanisms, we analyzed the protein expression levels of key signaling molecules in the left ventricular tissue of the seven experimental groups using the Western blot technique. The molecular markers analyzed included SMAD2, as well as Nf-κB p50. The expression levels of these molecules were systematically normalized to GAPDH, a constitutively expressed housekeeping protein, to ensure accurate quantification and comparative analysis.

**Figure 7:**
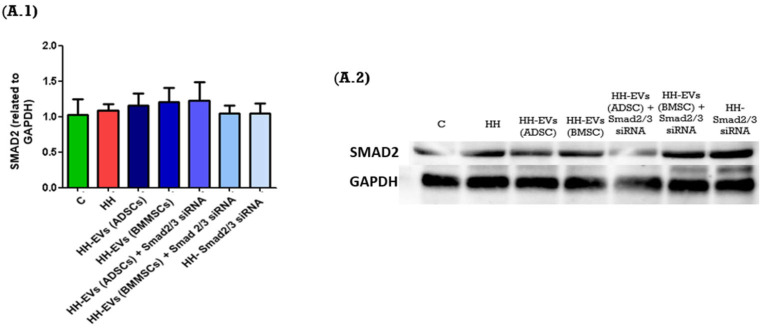

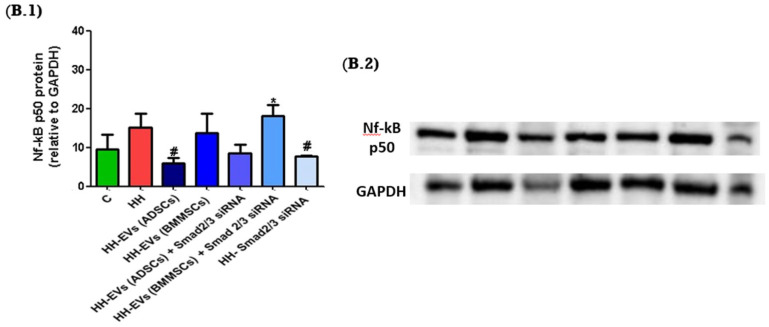
Western blot analysis results and representative images of the blots for the proteins analyzed, as follows: (**A.1**,**A.2**)—SMAD2, (**B.1**,**B.2**)—Nf-κB p50 and GAPDH in the left ventricle of the seven experimental hamster groups. The charts summarize protein levels in all investigated groups based on four independent experiments conducted after four months of diet and/or treatment. Data are presented as mean ± standard deviation (SD). Protein expression levels were normalized to GAPDH. Statistical significance is indicated as * *p* < 0.05 compared to the control group; and **^#^** *p* < 0.05 compared to the HH group. Two-way ANOVA, Bonferroni post-test was applied.

The results obtained showed similar results for the SMAD2 protein between the seven experimental groups (Figure 7(A.1,A.2)). However, for the Nf-κB p50 expression, an increase was observed for the HH group compared to the control group. Regarding the treated groups, a significant decrease was observed for the HH-EVs (ADSCs) and the HH-Smad2/3 siRNA groups (^#^ *p* < 0.05). Additionally, a reduction was also observed for the HH-EVs (ADSC) + Smad2/3 siRNA group, suggesting the capacity of this therapeutic approach to target the molecular mechanisms responsible for the onset of cardiac hypertrophy in atherosclerosis (Figure 7(B.1,B.2)).

These findings provide further evidence for the therapeutic efficacy of this approach.

## 4. Discussion

The present study sought to evaluate the therapeutic potential of EVs derived from ADSCs and BMMSCs with and without transfection with Smad2/3 siRNA and of Smad2/3 siRNA alone in a hamster model of atherosclerosis-associated cardiac hypertrophy. The findings of the study demonstrate that both morphological and molecular changes induced by an atherogenic diet and NaCl gavage can be significantly modulated by EV-based therapies. The study successfully tracked two critical aspects: the induction of atherosclerosis-associated cardiac hypertrophy through the administration of a specific diet, complemented by gavage, and the modulation of these effects by EV-based therapies and/or RNA silencing. Morphological changes in the hamster groups, such as structural, functional, and the modifications that occur at the molecular level (e.g., inflammatory markers and signaling pathways), were analyzed.

The present study was conducted using a well-characterized hypertensive–hyperlipidemic (HH) animal model [21] that reliably recapitulated key features of human atherosclerosis and cardiac hypertrophy. In this study, hamsters were subjected to an atherogenic diet that was enriched with 15% butter and 3% cholesterol. This was coupled with daily gavage of 8% NaCl. The results of the study showed that the hamsters developed significant dyslipidemia, hepatic steatosis, and marked alterations in left ventricular (LV) structure and function. The HH group demonstrated an elevated level of total cholesterol, LDL cholesterol, and triglycerides in their plasma. Concurrently, there was a reduction in LV cavity size and an increase in wall thickness. It has been confirmed through echocardiographic and biochemical analyses that the alterations observed are indeed consistent with the successful induction of atherosclerosis and cardiac hypertrophy in this particular model. Consequently, this provides a solid foundation for the evaluation of the therapeutic efficacy of EV-based interventions. In order to determine the moment when the atherogenic diet and the gavage induced the changes specific to atherosclerosis, as well as the efficiency of the therapeutic approach, we monitored the lipid profile of the seven groups of hamsters monthly. From the first month, we observed a milky appearance of the plasma of the hamsters in the HH group, as well as significantly increased levels of cholesterol, LDL cholesterol, and triglycerides. It is important to note that EV treatment, whether derived from ADSCs or BMMSCs, led to significant improvements in these plasma lipid levels. Notably, the combination of EVs with Smad2/3 siRNA led to reductions that were comparable to, or exceeded, those observed with EV treatment alone. These results are consistent with those of previous investigations, which have demonstrated that EVs can modulate lipid metabolism and attenuate the progression of atherosclerotic lesions by influencing cholesterol handling within cells [25]. Furthermore, the gross anatomical observations made at the conclusion of the experiment confirmed the presence of hepatic steatosis in the HH group. The reversal of these alterations in the treated groups indicates that EV-based therapy may help restore normal lipid distribution.

The approach adopted was to track the effects of diet, gavage, and treatment from the outside in. The range of issues tracked included the appearance of the hamsters, body weight, lipid profile, appearance of internal organs, heart weight, and the analysis of the molecular mechanisms involved.

Firstly, echocardiographic analysis revealed a significant decrease in LV cavity size accompanied by substantial thickening of the ventricular wall, in the HH group. These findings are consistent with those of earlier studies, which have demonstrated how atherogenic stimuli and hypertension contribute to cardiac remodeling, leading to pathological hypertrophy [15]. In contrast, for the treated groups, particularly those transfected with Smad2/3 siRNA, a significant enhancement of the shortening fraction was observed. This functional enhancement, despite only modest structural changes, suggests that EV-based interventions might restore myocardial elasticity and contractile function through paracrine effects. This finding is consistent with the observations reported in studies that have evaluated the use of EVs as modulators of cardiac repair, where improvements in functional parameters have been observed even in the absence of marked anatomical changes [48,49].

One of the first findings of our study in terms of the molecular mechanisms involved was the marked increase in TGF-β1 and AngII in the plasma of HH animals, reinforcing previous literature linking these molecules to the development of cardiac hypertrophy and atherosclerosis [17]. TGF-β1 is a multifunctional cytokine that exerts its effects by activating both SMAD-dependent and non-canonical signaling pathways. This leads to the transcription of genes that drive fibrotic remodeling and hypertrophic growth. This cytokine not only contributes to excessive extracellular matrix deposition but also promotes cardiomyocyte hypertrophy, thereby exacerbating the structural remodeling characteristic of atherosclerosis-associated cardiac hypertrophy [50]. AngII, conversely, is a pivotal effector of the renin–angiotensin system, exhibiting vasoconstrictive and pro-inflammatory properties. It has been demonstrated that AngII increases blood pressure and induces oxidative stress, both of which play critical roles in the initiation and progression of cardiac hypertrophy [51]. The stimulation of protein kinase C and other downstream signaling cascades by AngII serves to further amplify the hypertrophic response, thereby contributing to vascular inflammation and further plaque instability, thus leading to the progression of atherosclerotic lesions [52]. Treatments with EVs derived from ADSCs and BMMSCs with and without transfection with Smad2/3 siRNA and with Smad2/3 siRNA alone effectively reduced the plasma levels of TGF-β1, as indicated by the ELISA results. Conversely, the results observed in the plasma AngII levels exhibited slight variations. A notable observation is that in the HH-EVs (BMMSCs) group, the levels of AngII were found to be significantly higher compared to both the control group and the HH group. One potential explanation for this phenomenon is that in vivo models frequently exhibit heterogeneity, with some animals demonstrating greater sensitivity to diet and gavage compared to others within the same group. However, the results of plasma AngII levels are consistent with the findings in left ventricular tissue sections for M1 macrophage levels, where the HH-EVs (BMMSCs) group was the only one that did not show a significant decrease, and for Nf-κB p50 expression, where no differences were observed between the HH group and the HH-EVs (BMMSCs) group. A substantial decrease in plasma AngII levels was observed in the HH-EVs (ADSCs) and HH-EVs (BMMSCs) + Smad2/3 siRNA groups, with the most significant improvement documented in the group treated with Smad2/3 siRNA alone.

This finding indicates that EV-based therapies have the potential to directly target the molecular drivers of cardiac remodeling by reducing the signaling pathways responsible for fibrosis and hypertrophy. The significant reduction in these key mediators underscores the potential of EV-based strategies to modulate the pathophysiological processes underlying atherosclerosis-associated cardiac hypertrophy, aligning with studies that have targeted the TGF-β1 cascade to alleviate cardiac remodeling [53].

Inflammation and oxidative stress have been identified as central to the pathogenesis of both atherosclerosis and cardiac hypertrophy [54]. The immunohistochemical analysis conducted in this study revealed a marked increase in ROS levels in the HH group when compared to both the control and treated groups. These observations were aligned with the other measurements taken for specific inflammatory markers: MIF, ANF, COL1A, Cx43, α-SMA, CTN1, T lymphocytes, total macrophages, and M1 and M2 macrophages. Each of these markers has a well-established role in the pathogenesis of atherosclerosis and therefore monitoring them offers essential information about the effect of the chosen therapeutic approach. Specifically, MIF plays a critical role in regulating immune responses. Its increased expression has been demonstrated to promote the recruitment and activation of immune cells, thereby intensifying the local inflammatory response that is central to both atherosclerotic plaque formation and cardiac hypertrophy [55]. Similarly, ANF is released in response to increased cardiac wall stress and volume overload. Elevated ANF levels serve as an indicator of the compensatory mechanisms engaged by the myocardium to counteract hypertrophic stimuli [56]. COL1A is a key marker of fibrotic remodeling. Increased expression of COL1A has been observed to signify enhanced collagen deposition, which in turn leads to myocardial stiffness and reduced compliance. These features have been identified in advanced stages of cardiac hypertrophy and atherosclerosis [57]. Furthermore, Cx43 has been demonstrated to be essential for intercellular communication within the heart. Alterations in Cx43 expression have been demonstrated to disrupt electrical coupling between cardiomyocytes, with the potential to result in conduction abnormalities and arrhythmias, which in turn can further complicate cardiac dysfunction [58]. Furthermore, the present study documented significant infiltration of immune cells, including T lymphocytes and total macrophages. The equilibrium between the pro-inflammatory M1 macrophages and the anti-inflammatory M2 macrophages was disrupted, with a marked increase in M1 macrophages. This imbalance fosters a persistent pro-inflammatory environment, thereby amplifying the pathological remodeling processes underlying both atherosclerosis and cardiac hypertrophy [59]. The measurements obtained demonstrated a significant increase in the aforementioned markers and type cells for the HH group, thereby confirming the installation of the atherosclerosis-associated cardiac hypertrophy once again. In contrast, all treated groups demonstrated marked reductions in these inflammatory markers indicating the effectiveness of the therapeutic interventions in mitigating inflammation and tissue remodeling. However, an exception to this trend was observed for M1 macrophages; while improvements were noted in nearly all treated groups, the group receiving EVs-derived BMMSCs did not show a comparable reduction in M1 macrophage infiltration.

Finally, Western blot analyses provided additional insight into the mechanisms of action of EVs. The study assessed two of the multiple signaling pathways involved in the development of cardiac hypertrophy, focusing on the molecule chosen as a therapeutic agent, Smad2/3. Thus, we quantified the actors in the canonical signaling pathway, TGF-β1-Smad2/3, and the non-canonical one, TGF-β1-TAK1-Nf-kB, both of which have been implicated in fibrotic and inflammatory responses in the myocardium. The results obtained demonstrated differences between the control and HH groups. Specifically, the expression level of NF-kB p50 was elevated in the HH group. These results strongly suggest that the molecular mechanisms known to drive cardiac hypertrophy in the setting of atherosclerosis are activated in the HH model. Furthermore, the results obtained from the treated groups were both mixed and interesting. Specifically, for NF-kB p50, decreases were observed in the HH-EVs (ADSCs), HH-EVs (ADSCs) + Smad2/3 siRNA, and HH-Smad2/3siRNA groups. These reductions underscore the efficacy of the targeted therapeutic approach in attenuating the key signaling pathways that contribute to cardiac remodeling in atherosclerosis. While the findings did not demonstrate statistically significant differences for all molecules examined in the seven experimental groups, it can be concluded that the therapeutic approach to atherosclerosis-induced cardiac hypertrophy based on EVs derived from mesenchymal stem cells warrants heightened attention and further refinement.

## 5. Conclusions

Following the studies and experiments performed, we are able to formulate two main conclusions: (1) the hyperlipidemic diet doubled by daily gavage successfully led to the obtaining of an animal model that mimics atherosclerosis, as well as the associated cardiac hypertrophy. Physiological observations throughout the four months of the diet, along with the analysis of plasma parameters, confirmed the induction of dyslipidemia. Subsequently, echocardiographic measurements confirmed that the diet and gavage induced the reduction of the internal cavity, therefore the thickening of the left ventricular wall, specific to hypertrophy. Also, the structural changes aligned with the functional ones, in the HH group, with the shortening fraction being significantly reduced. The initial observations were subsequently confirmed by the experiments performed at the end of the diet period. Thus, both for inflammatory markers specific to atherosclerosis and associated cardiac hypertrophy (AngII, TGF-β1, MIF, ANF, CTN1, Cx43, α-SMA, COL1A, reactive oxygen species, T-cells, and macrophages) and for proteins involved in established signaling pathways (NF-kB p50), increased values were determined in the HH group compared to the control group.

(2) The second conclusion is represented by the effect of the therapeutic approach represented by EVs (ADSCs or BMMSCs) transfected or not with Smad2/3 siRNA as well as that with Smad2/3 siRNA alone. Thus, although the hamsters received the cholesterol-rich diet and gavage along with the treatment of the group they were part of throughout the entire experimental period, the observations and analyses performed during and after the end of their administration showed that EVs have the ability to delay the progression of the pathology, or even to regress it. The efficacy of these therapeutic interventions is evidenced by a number of factors. Firstly, improvements in cardiac function and reductions in lipid abnormalities have been demonstrated. Secondly, the interventions have been shown to mitigate inflammation, oxidative stress, and maladaptive signaling in the myocardium. The results obtained provide a compelling rationale for further exploration of EV-based therapies, especially mesenchymal stem cell-derived EVs, in the field of cardiovascular medicine. It is recommended that future studies concentrate on validating these findings in clinical settings and elucidating the molecular basis that confers the observed cardioprotective effects.

***Study limitations:*** As with all investigations that rely on animal models, the present study has several limitations that warrant explicit discussion: (1) the yield and composition of EVs can be influenced by factors such as confluence density, the number of passages, and the serum content of the culture medium. In order to reduce these sources of variation, both ADSCs and BMMSCs were used at passage 5 and incubated for 48 h in serum-free medium prior to the collection of EVs. (2) The EVs collected from the conditioned media of ADSCs and BMMSCs were stored at −80 °C until use, with a maximum storage duration of two to three weeks. However, experiments performed for their characterization by flow cytometry, electron microscopy, and zetananosizer showed that the number and structure of EVs were not affected by the freeze/thaw procedure. (3) Since EVs were administered systemically—either subcutaneously or via the retro-orbital sinus—a proportion of the vesicles is likely to accumulate in the liver, potentially reducing the dose that reaches cardiovascular tissues. (4) In some of our experiments, the treatment with non-transfected EVs showed more pronounced benefits than EVs transfected with Smad2/3-siRNA. This discrepancy is likely indicative of the multifactorial nature of atherosclerosis, and the observation that Smad2/3 does not necessarily exert a dominant influence across all phases or states of the disease. (5) With regard to the clinical applications of EVs, it is imperative to consider the potential adverse effects of such therapeutic interventions.

## Figures and Tables

**Table 1:** Clinical characteristics and biochemical parameters of the experimental groups evaluated after 16 weeks of dietary intervention and 14 weeks of treatment. In the HH group, the results obtained (marked in red) showed a significant increase in cholesterol, LDL cholesterol, HDL cholesterol, and triglycerides compared to the C group, indicating the onset of dyslipidemia. For the treated groups, the results (marked in green) showed significant reductions in plasma levels of cholesterol, LDL cholesterol, and triglycerides compared to the HH group.

After 16 Weeks of Standard Diet/Atherogenic Diet)	Control (n = 11)	HH (n = 18)	HH-EVs (ADSC) (n = 10)	HH-EVs (BMMSC) (n = 9)	HH–EVs (ADSC) + Smad2/3 siRNA (n = 5)	HH–EVs (BMMSC) + Smad2/3 siRNA (n = 6)	HH-Smad2/3 siRNA (n = 8)
Glycaemia (mg/dL)	116.02 ± 22.69	137.76 ± 48.25	137.97 ± 19.16	152.5 ± 21.81	108.81 ± 29.49	125.2 ± 16.1	133.96 ± 9.42
Cholesterol (mg/dL)	128.04 ± 14.47	641.66± 266.13	244.45 ± 60.6	300.83 ± 60.77	208.22 ± 37.7	267.1 ± 64.12	201.7 ± 36.47
Triglycerides (mg/dL)	93.42 ± 4.17	1073 ± 6.87	212.26 ± 3.42	175.43 ± 5.62	202.82 ± 4.89	217.31 ± 6.87	652.22 ± 55.75
HDL Cholesterol (mg/dL)	54.44 ± 6.47	109.73 ± 49.59	89.71 ± 29.72	82.67 ± 25.27	97.41 ± 3.97	90.24 ± 25.77	91.43 ± 23.52
LDL Cholesterol (mg/dL)	73.67 ± 4.69	635.43 ± 8.8	307.09 ± 7.23	303.18 ± 8.4	280.26 ± 2.3	364.18 ± 5.24	284.94 ± 43.91

## Data Availability

The original contributions presented in this study are included in the article/Supplementary Materials. Further inquiries can be directed to the corresponding author.

## Supplementary Materials

The following supporting information can be downloaded at: https://www.mdpi.com/article/10.3390/biom15101424/s1, Figure S1: Western blot representative original images of expression levels of SMAD2 and Nf-κB p50.
