# Supplementary figures and images for: Innovative Therapy with Stem Cell-Derived Extracellular Vesicles on Cardiac Hypertrophy in an Animal Model of Atherosclerosis; Elucidation of the Molecular Mechanisms Involved in the Repair Process
Source: Biomolecules. 2025 Oct 7;15(10):1424. doi: 10.3390/biom15101424 (PMC12563990; doi:10.3390/biom15101424)

Western blot representative images of expression levels of SMAD2 and Nf-B p50.

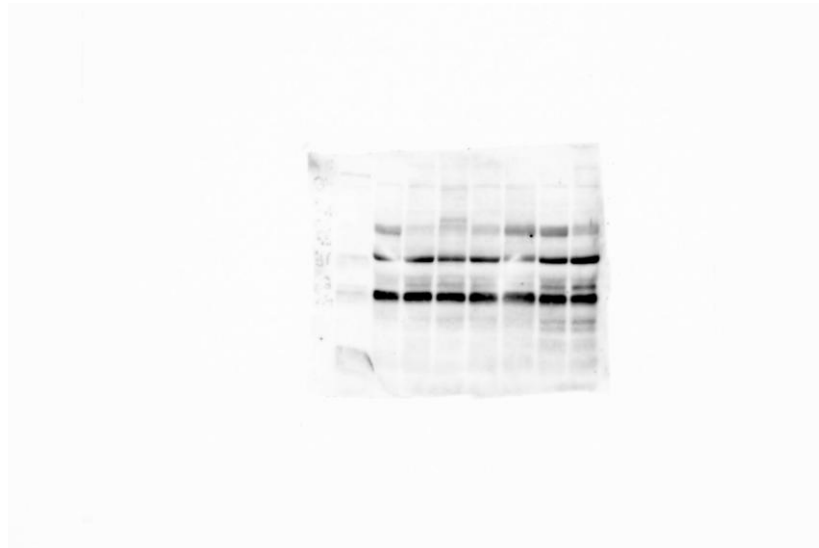

SMAD2

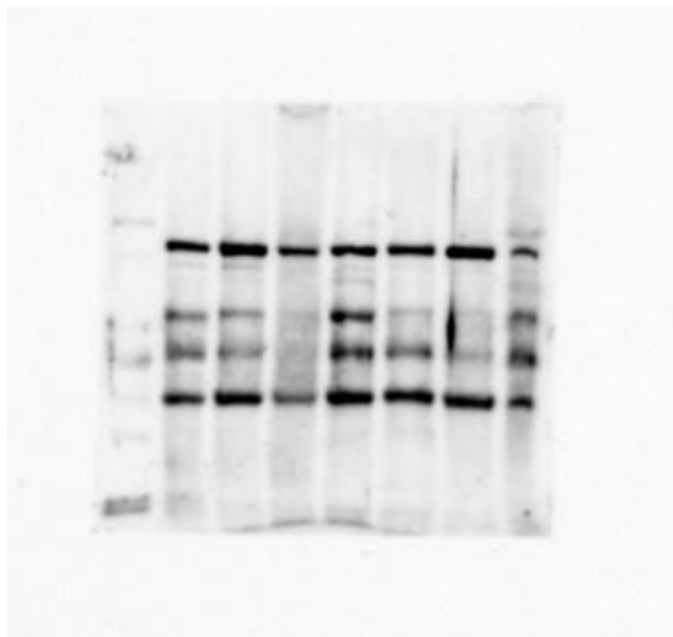

Nf-kb p50

Supplement: Supplementary file 1 [file biomolecules-15-01424-s001.zip › biomolecules-3852310-supplementary.pdf]
